# An open and transparent process to select ELIXIR Node Services as implemented by ELIXIR-UK

**DOI:** 10.12688/f1000research.10473.2

**Published:** 2017-03-20

**Authors:** John M. Hancock, Alf Game, Chris P. Ponting, Carole A. Goble

**Affiliations:** 1 Earlham Institute, Norwich Research Park, Norwich, UK; 2 Tatterdemalion Solutions, Marlborough, UK; 3 MRC Human Genetics Unit, The Institute of Genetics and Molecular Medicine, University of Edinburgh, Edinburgh, UK; 4 School of Computer Science, University of Manchester, Manchester, UK

**Keywords:** ELIXIR, ELIXIR-UK, e-Infrastructure, ESFRI

## Abstract

ELIXIR is the European infrastructure established specifically for the sharing and sustainability of life science data. To provide up-to-date resources and services, ELIXIR needs to undergo a continuous process of refreshing the services provided by its national Nodes. Here we present the approach taken by ELIXIR-UK to address the advice by the ELIXIR Scientific Advisory Board that Nodes need to develop “ *mechanisms to ensure that each Node continues to be representative of the Bioinformatics efforts within the country”. *ELIXIR-UK put in place an open and transparent process to identify potential ELIXIR resources within the UK during late 2015 and early to mid-2016. Areas of strategic strength were identified and Expressions of Interest in these priority areas were requested from the UK community. Criteria were established, in discussion with the ELIXIR Hub, and prospective ELIXIR-UK resources were assessed by an independent committee set up by the Node for this purpose. Of 19 resources considered, 14 were judged to be immediately ready to be included in the UK ELIXIR Node’s portfolio. A further five were placed on the Node’s roadmap for future consideration for inclusion. ELIXIR-UK expects to repeat this process regularly to ensure its portfolio continues to reflect its community’s strengths.

## Introduction

ELIXIR, the European infrastructure for life science data ^1^, is made up of individual Nodes, one for each of the organisation’s constituent members (21 as of 1st March 2017: Belgium, Czech Republic, Denmark, EMBL-EBI, Estonia, Finland, France, Germany, Hungary, Ireland, Israel, Italy, Luxembourg, Netherlands, Norway, Portugal, Slovenia, Spain, Sweden, Switzerland and the UK), and a coordinating hub. The individual ELIXIR Nodes provide the services and resources that support the five pillars of ELIXIR (Compute, Tools, Data, Interoperability and Training infrastructures). ELIXIR Services can be data resources, tools, and services, such as interoperability services or training centres.

ELIXIR nodes need to be able to evolve their contributions to ELIXIR by bringing new services and resources. ELIXIR identifies two types of service: Node-funded services, which are funded nationally and are contributed to ELIXIR from a national Node; and Commissioned Services, which are funded by ELIXIR as a whole via the ELIXIR Hub. In some ELIXIR Nodes, Node-funded services receive funds through their national Nodes; in the case of the UK’s Node, ELIXIR-UK, resource funding is through direct grant funding to resources and services from the national funders. In ELIXIR terms, these are still labelled as “Node-funded”. The process described in the present article was set up to identify Node-funded services and resources for ELIXIR-UK. In this context it should be noted that the services a Node can contribute to ELIXIR may fall under any one of ELIXIR’s five platforms: tools, data, compute, interoperability and training. ELIXIR-UK took a strategic decision to exclude potential compute resources from this process, at least initially, pending national commitment to contribute compute resources to international programmes. ELIXIR sets high standards for the services it provides. Consequently, nodes need to take full account of these requirements when selecting and proposing their services, which are ultimately judged for suitability by the ELIXIR Scientific Advisory Board (SAB) and Board of ELIXIR (see the online ELIXIR Handbook for more detail).

ELIXIR-UK was established in September 2013, and as its first contribution to ELIXIR took on a thematic focus, namely of coordinating training activity. More recently, it has sought to expand its remit. To address the SAB’s recommendation that Nodes put in place “ *mechanisms to ensure that each Node continues to be representative of the Bioinformatics efforts within the country”.*

The UK’s bioinformatics landscape is diverse with strengths across the range of ELIXIR’s activities. UK bioinformatics research is primarily supported either by grant funding to research groups based in Universities or to longer-term, rolling funding to groups in research institutes. Although there are some dedicated funding sources for bioinformatics resources (notably the BBSRC’s Bioinformatics and Biological Resources Fund and the Wellcome Trust’s Biomedical Resources Development Grants) much development, particularly of bioinformatics tools, is carried out as part of 3-year or shorter research grants. Long-term sustainability of new resources is therefore an ongoing issue for the UK bioinformatics community, as it is in other territories. UK funders have a range of interests and a commitment to fundamental research but some areas are particularly well-supported: health and welfare-related research receives dedicated funding via the MRC and from the Wellcome Trust, and BBSRC focusses significant resource into research relating to food security (primarily agricultural animals and plants). The UK has particular and long-standing strengths in genomics and structural biology. It is worth noting that the European Bioinformatics Institute (EBI), which is located in the UK, is not part of the UK Node of ELIXIR but it is a frequent partner in UK-funded research grants. Many UK resources likely to become part of the UK Node have established links with the EBI.

To reflect this diversity, and its perceived strengths, ELIXIR-UK developed a process to choose new services and resources to add to its existing portfolio that would:

• Reflect national strengths and priorities in bioinformatics
• Engage its national community; and
• Be robust, transparent and open so that its community would regard it as fair and it could continue to be applied to allow the Node to develop over time.

## Process overview

**Figure 1.**
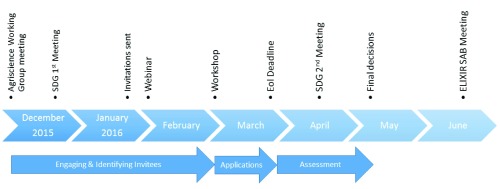
Summary timeline of the ELIXIR-UK Node Expansion process. Bulleted items represent milestones in the process; arrows below represent the three main phases: engaging the community and identifying potential applicants to invite (the call was also open to non-invited applications); receipt of applications; and assessment of applications by the SDG including feedback and iteration with applicants.

As illustrated in Figure 1, the process implemented by ELIXIR-UK went through seven key phases, which are expanded on in the following sections:

1. Strategic prioritization
2. Identifying possible candidate resources
3. Establishing a Scientific Development Group to provide external advice on the suitability of candidate resources
4. Establishing assessment criteria
5. Engaging the community
6. Assessing Expressions of Interest
7. Finalising a new portfolio

## Strategic prioritization

The requirement to ensure that each Node continues to be representative of the Bioinformatics efforts within the country could be seen as open ended, and thus could ultimately lead to an ill-focussed collection of resources and services. To avoid this, ELIXIR-UK identified a set of priority areas within which to focus submissions to the process (in this context, and when discussing any other decisions made by ELIXIR-UK, note that decisions were formally made by the Node Executive Committee which consisted of the Head of Node, Deputy Head of Node, Node Coordinator, Technical and Training Coordinators and three rotating members; the constitution of the Node will change in 2017 and this Executive Committee will be replaced by Management and Steering Committees which will take on final decision-making responsibility). These were initially identified by discussions within the Node and were refined by discussion with the Node’s funding organisations (Biotechnology and Biological Sciences Research Council [BBSRC], Medical Research Council [MRC] and Natural Environment Research Council [NERC]) and with the Scientific Development Group (SDG), which was a community body set up by the Node that is tasked with identifying new node resources (see below).

As a consequence of these discussions, Expressions of Interest (EoIs) were invited in the following priority areas, identified as being of high strategic importance within the UK:

• Human clinical and health ‘omics and related areas in health informatics
• Agricultural ‘omics and related data resources
• Image informatics (including atlases)
• Structural bioinformatics
• Technical infrastructure for interoperability and training including standards

## Identifying possible candidates

ELIXIR-UK aimed to reconcile two potentially conflicting drivers in developing its expansion process. Firstly, it wanted to be as open to the UK bioinformatics community as it could. This is an ongoing challenge because a) ELIXIR has incomplete brand recognition within the UK community, and b) is not well-regarded by some, being seen either as a closed club or unproductive. Secondly, the Node wanted to ensure it received Expressions of Interest from potential services and resources that were demonstrably of high value to the international life sciences community. To address these requirements, the Node approached the recruitment of potential candidates in two ways. Firstly, it publicised its “Node Expansion” process well in advance using its web site, Twitter and word-of-mouth. Secondly, it sent targeted emails to potential candidate resources. These were identified using a variety of inputs:

• Brainstorming by members of the existing node
• Setting up a specific working group - on Agriculture-related data - for an area that was not well-represented in the current node
• Additional suggestions from the SDG (see next section) and funders

## The Scientific Development Group

The key body in the Node’s expansion process was the SDG. This was set up by the Node to evaluate EoIs to join the Node against a set of published criteria (see below). This group was also involved in refining those criteria and providing suggestions of resources to be invited to provide EoIs.

The membership of this group was based on suggestions from within the Node and from its funders. The members of the group are listed in the acknowledgements section. The group’s composition reflected the priority areas identified for the expansion, geographic spread, and the inclusion of at least one industry and at least one overseas representative. The Chair was chosen for his experience as a senior officer of a UK funding agency and knowledge of appropriate processes for activities of this kind. For the record we note that the group did not have an appropriate gender balance (it was 100% male). This is a defect we intend to remedy in future.

## Assessment criteria

Over time ELIXIR has been evolving both its classification of resources and its criteria for selecting them. During the period of the UK Node’s expansion process these definitions and criteria continued to evolve. The assessment criteria developed by ELIXIR-UK were developed by internal discussion within the Node’s Executive and in discussion with the SDG and were also discussed informally with the leaders of Work Package 3 of the EXCELERATE programme (Jo McEntyre and Christine Durinx), as their criteria developed in parallel (the WP3 criteria were not available for consideration by the Node in detail when it was developing its criteria but the WP3 leadership confirmed to us that our criteria were similar to theirs). EXCELERATE Work Package 3 aims to identify key data resources across Europe and support the linkages between data and the scientific literature. The final set of criteria, which were provided to applicants as an openly shared Google document, were:

1. Alignment with the five ELIXIR infrastructure themes (data, tools, compute, interoperability, training)
2. Strong complementarity to the 2014-18 ELIXIR programme
3. Complementarity to ELIXIR-UK strategic themes
4. Potential for cross-Node collaborations
5. Provision of comparable impact to existing ELIXIR resources from other Nodes already accepted by the ELIXIR SAB
6. Resource contribution to wider EU infrastructures and integration
7. Ability to interoperate with other ELIXIR resources
8. Evidence of community outreach and adoption
9. Leadership in data stewardship within a community
10. Evidence of long-term sustainability

To facilitate applicants demonstrating that their resources fulfilled these criteria, an Expression of Interest template form was provided (see Supplementary file 1). EoIs were received on a confidential basis.

The criteria developed by EXCELERATE Work Package 3 have subsequently been finalised and form the basis of the ELIXIR process for selection of Core Data Resources ^2^.

Criteria for training resources were necessarily different for those for data, tools and interoperability resources. Criteria 1-4 applied as for other resources as these concern compatibility with ELIXIR’s strategy and activities. Criterion 5 could not be directly applied as other Nodes did not at that time offer training centres as resources, but the principle of comparable quality to other ELIXIR training activities was applied. Criterion 6 was relevant in that training resources needed to have an outward-looking approach and should not only provide training locally. Criterion 7 was not directly applicable but Criterion 8 was a key indicator of quality. Criterion 9 was relevant where centres showed leadership, e.g. in the ELIXIR Training Platform or in coordinating training activities more widely. Sustainability of training activities (Criterion 10) is generally different for training activities which (in the UK) do not generally receive strategic funding to provide training but rather are expected to be self-funding. A record of successful self-funding over a period of time was therefore expected.

## Engaging the community

As outlined, it was important to ensure community buy-in to this process (in order to ensure that the Node was able to engage sufficient high quality resources) and at the same time it was important to be sure that community members who might be interested in participating in ELIXIR-UK were aware of what was required and the expectations that would be placed on them as ELIXIR-UK Node resources. Formal community engagement took place in two phases: a webinar, led by the Head of Node (CAG) and Node Coordinator (JMH), in February 2016 and a workshop, hosted at the Wellcome Trust building in London, in March 2016. The aim of the webinar was to introduce ELIXIR and ELIXIR-UK and the rationale behind the node expansion process. The aim of the workshop was to introduce and discuss the assessment criteria in detail, so that potential applicants could be clear as to what was required. The presentation given at the workshop is available via Slideshare. At this stage a deadline was set for the receipt of Expressions of Interest by the Node. It is worth noting that the deadlines for the process were tight: EoIs were requested by the end of March 2016 and the assessment meeting took place at the end of April with some iterations taking place in May. We were fortunate in being able to run such a tight schedule due to a) clear and lightweight requirements for the EoIs; b) what we believe to be clear and effective communications; and c) motivated applicants and SDG members.

One result of being open to community input was that the original scope of the call – for data, tools and interoperability resources – was broadened to include also training resources. This was done because existing members of the Node who were providing training wished to be recognised on the same basis as providers of more technical types of resource. The training resources considered during the process were of two types: providers of training courses and training infrastructure.

## Assessment of Expressions of Interest

EoIs were assessed by the SDG against the published set of criteria. To facilitate assessment of EoIs, three group members were allocated to each EoI (18 were submitted). The three members were asked to score EoIs from 1 to 4: 1 = ready for inclusion in ELIXIR (“infrastructure ready”); 2 = further discussion or clarification needed; 3 = not ready, but suitable enough to be placed on a roadmap for future inclusion; 4 = not suitable. The assessments for each EoI were introduced by one member of the group leading on to an open discussion. Representatives of the Node funders and the ELIXIR-UK executive observed the meeting to give advice on strategic alignment. EoIs were given a consensus final score using the same scale as before, with a score of 2 in this case representing the need for further clarification of issues raised by the group. Resources given a 2 rating were asked for further information, which led to their final score being revised upwards or downwards in a subsequent iteration.

Four of the five strategic areas that were laid out initially received three or four Expressions of Interest. The one receiving fewer EoIs was the theme on Image Informatics, for which only one proposal was received (although we note that the Biomedical Atlas Centre was explicitly formed to address this call). Although we specified strategic priorities we also informed potential proposers that we were open to proposals outside of these priority areas. Consequently, and reflecting ELIXIR-UK’s involvement with the ELIXIR Training platform, we received enquiries as to whether we would also consider EoIs from training centres. In the spirit of supporting the widest possible engagement within ELIXIR-UK we agreed to this suggestion and received three proposals for training centres, all of which were accepted.

A point to note is that a number of the resources that were considered are not UK-only – many are joint activities that span different countries, some with their own ELIXIR Node. This was a topic of some discussion in the SDG meetings as ELIXIR-UK is intended to represent UK contributions. On the other hand, excluding international activities per se could have the effect of preventing some valuable resources from becoming part of ELIXIR. The SDG decided to include resources if they could make the case that they were primarily based in the UK or the UK contribution was significant and the resource was unlikely to be put forward by another ELIXIR Node.

Table 1 contains some comments on how each of the criteria were applied during the assessment process.

**Table 1.** Notes on the ways in which the SDG applied the general requirements of the ten assessment criteria.

Criterion	Comments
1. Alignment with the five ELIXIR infrastructure themes (data, tools, compute, interoperability, training) 2. Strong complementarity to the 2014-18 ELIXIR programme	These two criteria supplied a high-level test as to whether proposals described the kinds of resources that could be envisaged as contributing to ELIXIR a) as bioinformatics resources and b) as fitting within the realm of activity of ELIXIR. As an example, a training centre focussing on in biochemistry wet lab techniques would pass criterion 1 but not criterion 2
3. Complementarity to ELIXIR-UK strategic themes	This more stringent test assessed whether there was clear complementarity between the data, tools, interoperability or training provided by the resource and the strategic themes identified by ELIXIR.
4. Potential for cross-Node collaborations	Potential resource were assessed as to whether they currently interacted with resources provided by other Nodes (noting that information on this was only sketchy when the assessment was carried out) or more broadly if they interacted with resources in other ELIXIR countries
5. Provision of comparable impact to existing ELIXIR resources from other Nodes already accepted by the ELIXIR SAB	This criterion was used to gauge the level of international recognition resources had, and how important they were to their communities. High importance to a small community was considered as important as reach to a wider community. One aspect of this assessment was usage metrics. Use of metrics currently suffers from lack of consistency between resources; generally successful data resource achieved the equivalent of 10,000 unique users per annum.
6. Resource contribution to wider EU infrastructures and integration	The degree to which resources could claim to contribute to wider EU infrastructures was generally limited. Generally responses to this criterion represented aspirations to interact more as part of ELIXIR.
7. Ability to interoperate with other ELIXIR resources	This was a test of whether technical resources used or provided metadata standards or APIs that were open to external users including ELIXIR resources, or intended to move in that direction. Technical standards for ELIXIR were in their earliest stages when this assessment was carried out so a clear mapping to Interoperability Platform recommendations was not possible.
8. Evidence of community outreach and adoption	Two main aspects were considered under this criterion – the amount of community usage and recognition, and the outreach activities the resources provided, including training.
9. Leadership in data stewardship within a community	This was more relevant in some cases – when a resource was a major resource or repository for a single community – than for others where their resource provision was more cross-cutting.
10. Evidence of long-term sustainability	Evidence of sustainability considered was twofold – firstly the track record of past funding and current funding commitment and secondly any institutional commitment to support the resource. At least one resource’s case was weakened by lack of current funding. Shifts in institutional funding from UK funders mean that there is little opportunity for stable, long-term funding from institutes even when these are funder-branded because of (typically) 5-year funding renewals.

## Results of the assessment

The outcomes of the assessment are summarised in Table 2. Nine EoIs were considered to be infrastructure ready (score of 1) on the first pass of assessment, and a further five were asked for more detail on their proposal (score of 2).

**Table 2.** Summary of outcome of the Scientific Development Group deliberations. The table gives numbers of proposals classified as 1 (ready for inclusion in ELIXIR (“infrastructure ready”); 2 (further discussion or clarification needed); 3 (not ready, but suitable enough to be placed on a roadmap for future inclusion); 4 (not suitable).

Rating	After panel assessment	After iteration
1	10	13
2	5	1 ^*^
3	4	5
4	0	0

* *In this case the group were unclear whether the proposed resource could be included in the Node’s offering. This case was put forward to the ELIXIR SAB for further input who recommended it be placed in Category 1.*

An iteration of discussions with resource scientists allowed questions raised by the SDG to be considered further. Where these were answered satisfactorily, resources were moved up to infrastructure-ready status. Otherwise they were put on the roadmap or, in one case, referred to the ELIXIR SAB for further comment (in this latter case, SAB guidance subsequently resulted in it being accepted as infrastructure ready).

After ratification by the ELIXIR-UK executive and notification to the ELIXIR Hub, highly rated resources were included directly into the Node’s portfolio and were included in the Node Application presented to the ELIXIR SAB in June 2016 and the ELIXIR Board in November 2016. Others were placed on the Node’s roadmap for possible future inclusion.

The services and resources selected as ready for immediate inclusion are listed in Table 3.

**Table 3.** Resources judged to be ready for inclusion in ELIXIR at the end of the assessment process. Resources are classified by strategic themes within ELIXIR-UK.

Name	Strategic theme	URL
Ensembl – farmed and domesticated animals ^3^	Agri-food data	www.ensembl.org
Pathogen Host Interactions Database (PHI-base) ^4^	Agri-food data	http://www.phi-base.org
Biomedical Atlas Centre	Gene expression atlases	http://emouseatlas.org/, http://hudsen.org/, http://echickatlas.org/
IUPHAR/BPS Guide to Pharmacology ^5^	Human health & disease data	www.guidetopharmacology.org
BioSharing ^6^	Interoperability services	https://biosharing.org
InterMine ^7^	Interoperability services	http://www.intermine.org, https://github.com/intermine/intermine
ISA Tools & Commons ^8^	Interoperability services	http://www.isa-tools.org http://www.isacommons.org
CATH-Gene3D ^9^	Protein structure & function	http://www.cathdb.info/
Jalview and the Dundee Resource for Sequence Analysis and Structure Prediction ^10^	Protein structure & function	http://www.jalview.org www.compbio.dundee.ac.uk/jpred
Phyre2 ^11^	Protein structure & function	www.imperial.ac.uk/phyre2
Birmingham Metabolomics Training Centre	Training	http://www.birmingham.ac.uk/facilities/metabolomics-training-centre/ index.aspx
Cambridge Bioinformatics Training Programme	Training	http://bioinfotraining.bio.cam.ac.uk/ http://training.csx.cam.ac.uk/bioinformatics/Event-timetable
Edinburgh Genomics Advanced Training in Bioinformatics	Training	https://genomics.ed.ac.uk/services/training
TeSS (Training e-Support System)	Training	https://tess.elixir-uk.org/

*References for the databases/tools have been added where available.

## Future activities and conclusions

We believe that the process outlined here was open, transparent and fair. We carried out a survey of proposers, SDG members and other members of ELIXIR-UK which strongly supported this view. It also identified two areas that we will need to pay attention to in future: the quality of feedback to proposers, especially unsuccessful ones (i.e. those which are roadmapped), and how widely the exercise is advertised to attract applicants. We note that the “success rate” of the process was high. No resources were rejected outright and more than 70% were promoted immediately to the Node’s portfolio. This does not reflect a lax process, but is likely to have had a number of contributing factors, including:

• The fact that this was the first call of this kind meant that the Node could call on a number of outstanding, internationally-acknowledged resources. The resources placed on the roadmap were generally also well regarded, but usually in an early phase of their development. Our expectation is that most of these will be recognised as Node-funded resources in future.
• There was a clear explanation and open presentation of the high standards expected of successful resource. Therefore, it is likely that only resources that considered they had a realistic chance of success after the webinar and workshop put their names forward. Consequently, we did not receive any truly speculative proposals.

Another aspect of the process we outline here is the short time period over which it was carried out. In particular, resources were only given four weeks to submit EoIs. A number of features of the process facilitated this: clear timelines, clear guidance as to what was required, the availability of a template for EoIs that helped proposers to compile their EoIs, and lightweight requirements for completing EoIs, which were nevertheless sufficient to allow the SDG to carry out its work effectively. Engagement at a senior level by both the Node and proposers was also important. It was also important to organise meetings, especially of the SDG, sufficiently ahead of time to allow members to both assess the EoIs and attend the meetings, either in person or remotely.

We expect this process to be readily generalizable to other ELIXIR Nodes who wish to evaluate candidate resources. One limiting factor for smaller Nodes could be critical mass of potential reviewers so that it might be necessary to bring in a higher proportion of reviewers from outside the country concerned (the UK is fortunate in having a large pool of potential assessors not currently involved in ELIXIR; this could become more problematic as more UK groups participate in ELIXIR-UK. As an example, one of our SDG members in 2016 was PI of a resource that was selected for inclusion. The applicant was asked to leave the room while their resource was discussed as is normal in UK grant assessment panels but clearly minimising overlap between applicants and assessors is critical). This process also benefitted from close interactions that exist between the Node and its funders, which allowed ELIXIR-UK to have a clear picture of its funders’ priorities in setting its own strategic priorities. On the other hand Nodes might decide to address ELIXIR-wide or other sets of priorities instead of or as well as national priorities.

To maintain and continue to improve the Node’s alignment with UK research strengths, it plans to hold regular refresh exercises to introduce new resources into the Node. Plans for how this will be done are currently under development. To pursue this process we expect that we will need to develop community engagement in the specific priority areas, so that potential proposers are primed. To complement this it will be necessary to continuously evaluate the performance of the Node resources that have been selected to ensure that they continue to meet the Node’s quality guidelines. ELIXIR-UK is in the process of establishing a new constitution under which quality assessment of the Node’s offerings to ELIXIR will be supervised by its Science and Industry Advisory Board. It is likely this body, or a subcommittee of it, will be tasked with carrying out periodic assessments of delivery by Node resources.

An important aspect for the future will be supporting roadmapped resources in achieving full Node resource status and possibly, in future, status as ELIXIR Core Data Resources. The requirements for this will be specific for different resources but two aspects we have identified are the ability to discuss potential integration into ongoing ELIXIR activities (in particular in light of funding for networking activities being limited for many resources) and improved technical capabilities, including APIs and use of ELIXIR-compliant standards to facilitate technical integration into the ELIXIR data ecosystem. We are endeavouring to establish funding streams to support developments of this kind.

## Supplementary Material

Supplementary file 1: Applicants expression of interest template form.
