# Supplementary material for: An open and transparent process to select ELIXIR Node Services as implemented by ELIXIR-UK
Source: F1000Res. 2017 Mar 20;5:ELIXIR-2894. Originally published 2016 Dec 21. [Version 2] doi: 10.12688/f1000research.10473.2 (PMC5265702; doi:10.12688/f1000research.10473.2)
Supplement: Supplementary file 1 [file f1000research-5-12033-s0000.tgz › f99dba35-0a38-4039-8ba4-24b06e5f6898.docx]

EXPRESSION OF INTEREST TEMPLATE FOR RESOURCES WISHING TO BECOME ELIXIR-UK NODE RESOURCES

[ELIXIR](https://www.elixir-europe.org/) is a Europe-wide infrastructure for Life Science data. It operates through a set of national Nodes coordinated by a Hub located at Hinxton. National nodes contribute activities, generically known as Resources, to the ELIXIR network. ELIXIR Node Resources are infrastructural resources that provide a long-term, sustainable and high quality service to the bioinformatics community. ELIXIR Resources fall within one of five themes: Data, Tools, Compute, Interoperability and Training. The Resources contributed by a national Node are set out in a Node’s Application, and must be approved by ELIXIR’s SAB and Board. Node Resources are expected to be financially sustainable, aligned to the ELIXIR platform of activities, and of high international impact.

The initial Node Application from the UK, to create and deliver [ELIXIR-UK](http://elixir-uk.org/), was restricted to the Training theme. However, the Node wishes to better represent the broad base of UK bioinformatics by expanding its remit across the five ELIXIR themes. (Note that, in the context of ELIXIR, EMBL-EBI is not a UK institution). To accomplish this, ELIXIR-UK is establishing a representative and diverse panel, chaired by Alf Game, to assess possible resources to be put forward as Node Resources to the ELIXIR SAB in late 2015. At this stage ELIXIR-UK particularly invites proposals within the Interoperability and Training themes. In addition ELIXIR-UK has identified four areas of strength for the UK that it expects to emphasise in its revised remit. These are:

- Human clinical and health genomics and related areas in health informatics,
- Agricultural omics and other data resources including those on an environmental scale,
- Image informatics (including atlases), and
- Structural bioinformatics.

In order to assess potential Node Resources, candidate resources are asked to complete the following template with the relevant information in as many areas as possible. Each of the main areas in the template corresponds to an aspect of the assessment process, although it is not essential that all be completed. The panel will be assessing resources based on their alignment and complementarity to ELIXIR themes, their impact and level of usage, and their sustainable nature based on past record and future plans. It is expected that around ten resources will be put forward as new UK Node Resources but this is not a fixed number.

This 2016 process may be the first of a series of annual calls, depending on the level of interest and strategic considerations.

PLEASE COMPLETE THE FOLLOWING TEMPLATE IN NO MORE THAN FOUR A4 PAGES.

Completed proposals should be returned by email to John Hancock (ELIXIR-UK Node Coordinator) at john.hancock@tgac.ac.uk by 5 pm on 31st March 2016. The panel may contact proposed resources for additional information if it is felt this is required.

Proposers may annex additional material to their proposals (*e.g*., letters of support), but please keep these to a minimum.

Please note that normal Research Council procedures with respect to conflict of interest between proposals and panel members will be adhered to during panel discussions.

| RESOURCE NAME: |
| --- |
| WEB ADDRESS: |
| LEAD INVESTIGATOR(S): |
| UK INSTITUTION(S) OF INVESTIGATOR(S): |
| CONTACT EMAIL(S): |
| 1. Alignment with one or more of the five ELIXIR themes (data, tools, compute, interoperability, training). It is to be expected that any resource put forward by the node will fit under at least one of these five categories. |
| 2. Complementarity to the 2014-18 [ELIXIR programme](https://www.elixir-europe.org/about/elixir-programme-2014-2018). Resources should be directly relevant to work packages in [EXCELERATE](https://www.elixir-europe.org/news/elixir-accelerates-major-horizon-2020-funding) or to other ongoing ELIXIR activities. |
| 3. Complementarity to ELIXIR-UK strategic themes: Human clinical and health omics^^[[1]](#footnote-1)^^ and related areas in health informatics; Agricultural omics and related data resources. These may include data generate up to and including an environmental scale; Image informatics (including atlases); Structural bioinformatics; Technical infrastructure for interoperability and training including standards. Note that Innovative resources in other areas with the potential to be of international importance may also be considered. |
| 4. Possibility of cross-node collaborations within ELIXIR. Identify opportunities for collaborations with ELIXIR activities in other Nodes. If such collaborations are already underway or concretely planned, please provide evidence. |
| 5. Resource impact. This should be comparable to existing ELIXIR resources from other Nodes already accepted by the ELIXIR SAB. See [this web page](https://www.elixir-europe.org/services) for a list of current services (resources). |
| 6. Resource contribution to wider EU infrastructures and integration. |
| 7. Ability to interoperate with other ELIXIR and global resources, e.g. via a public API and the use of accepted standards and terminologies, steps being taken to achieve this status or plans in place to do so. |
| 8. Evidence of community outreach and adoption. |
| 9. Leadership in data stewardship within a community, for example but not necessarily as a community database for a particular research community. |
| 10. Evidence of long-term sustainability. Evidence of as many of the following as possible:   1. Previous funding track record. Provide dates, amounts and sources of previous and current funding streams (applications in progress may be included here or under c) 2. Strategic commitment from host institution, funder or other organisation. indicate the nature and extent of institutional commitment to the future provision, maintenance and/or development of the resource. Formal confirmation of these from institutional authorities is not required at this stage but may need to be provided at a later stage of assessment. 3. Plan for future funding. 4. Indicate the personnel and physical resources currently committed to running and maintaining the resource and the minimum necessary to do so. |

1. omics data is high volume molecular data, for example but not restricted to genome sequence, the transcriptome, proteome and metabolome [↑](#footnote-ref-1)
