# Supplementary material for: An open and transparent process to select ELIXIR Node Services as implemented by ELIXIR-UK
Source: F1000Res. 2017 Mar 20;5:ELIXIR-2894. Originally published 2016 Dec 21. [Version 2] doi: 10.12688/f1000research.10473.2 (PMC5265702; doi:10.12688/f1000research.10473.2)
Supplement: Peer review history

# Peer review: Referee response for version 1

**DOI:** 10.5256/f1000research.11283.r18696

**Published:** 2017-01-23

**Authors:** Janet Kelso

**Affiliations:** 1 Department of Evolutionary Genetics, Max Planck Institute for Evolutionary Anthropology, Leipzig, Germany

This manuscript describes the process whereby the UK node of ELIXIR identifies and prioritises services for inclusion in the node.

 It is an important contribution as it is the first time a strategy for service identification and prioritization has been documented for an ELIXIR nodes, and it may be able to be generalized for use by other nodes of the ELIXIR network. It would be good if the authors could comment on the extent to which this approach is generalizable to other nodes, and on whether there are aspects that are unique to the UK node.

  

- The authors should clarify early on that the ‘services’ that ELIXIR nodes may offer may include any combination of software, data resources and training. This is implied, but may not be completely clear to readers unfamiliar with ELIXIR.

- The authors focus primarily on a process to include new services to the UK ELIXIR node. It would be good to add a brief section that discusses long-term evaluation of existing services in order to assess ongoing quality and relevance, and to plan for retirement of services as required.

- There are places where it is unclear who represents “the Node” eg: “These were initially identified by discussions within the Node” and “Brainstorming by members of the existing node”. It may be useful to explain who is responsible for the Node and decisions taken by “the Node”, and therefore for the strategic prioritization and evaluation.

- Could the template for EoI be made available for other nodes wishing to follow a similar process?

- It would be useful to expand briefly on what “Work Package 3 of the EXCELERATE programme,” is, so that a reader unfamiliar with ELIXIR can understand the relevance.

- The authors conclude that the process was transparent and fair. Has there been any community-feedback on the process? It would be interesting to know how well-accepted the process has been, and whether there are any suggestions for improvement.

- It would be useful to include information about the matching of the EoIs received with the strategic priorities that were identified. Were applications received in all priority areas? Are there areas that are not yet represented? How do new services that have been included relate to those that were already existing within the node, and also to services provided by other nodes (and was this a consideration in the evaluation process?)

- Is there any plan to support service proposals that were assigned to the roadmap for future inclusion?

I have read this submission. I believe that I have an appropriate level of expertise to confirm that it is of an acceptable scientific standard, however I have significant reservations, as outlined above.

# Peer review: Referee response for version 1

**DOI:** 10.5256/f1000research.11283.r18698

**Published:** 2017-01-09

**Authors:** Alfonso Valencia

**Affiliations:** 1 Structural Biology and Biocomputing Programme, Spanish National Bioinformatics Institute (INB), Spanish National Cancer Research Centre (CNIO), Madrid, Spain

The report describes the process of selection of the UK-ELIXIR xxxx to be presented to the ELIXIR SAB.

 The report is very informative and being the first node that describes in detail their national process, it has the potential to be very useful for nodes in other countries.

 I have a number of suggestions of additional information that is mentioned in the text but not explicitly included.  

 Composition of the Scientific Development Group (SDG)

 A figure with the time line of the process that could be integrated with the Fig. 1.

 The template used for the EoIs.

 The EoIs submitted by the selected resources (if possible)

 I also have a few other questions that may help to clarify specific aspects.

 Three training resources were finally selected but in the description of the UK-ELIXIR strategic items training is not described separately (´Technical infrastructure for interoperability and training including standards). If possible, it may help to clarify what was understood by training in the strategic items and how it is different, or not, of the technical infrastructure.

 If it would be possible to give some additional explanation to some of the ' final set of criteria’ that may really help others. One possible way might be providing examples of answers provided by some of the applications.

 It is not very clear how the 'ELIXIR criteria for the selection of Core Data Resources' was incorporated in the process. Given the importance of the ELIXIR guidelines for future similar processes this point could be quite relevant.

 Finally, even if I realise that this might be considered outside the scope of this paper,  what will be really interesting is to include a short explanation of how the selected resources fit each one of the selection criteria, at least at some level of detail.

I have read this submission. I believe that I have an appropriate level of expertise to confirm that it is of an acceptable scientific standard, however I have significant reservations, as outlined above.

# Peer review: Referee response for version 1

**DOI:** 10.5256/f1000research.11283.r18697

**Published:** 2016-12-30

**Authors:** Christine Durinx

**Affiliations:** 1 SIB Swiss Institute of Bioinformatics, Lausanne, Switzerland

The article is clear and well written. The figure and tables support the content.

 Some suggestions, mainly to improve clarity:

*Introduction*

 - It would be helpful to mention that the ELIXIR Services can be data resources, tools, and services.

 - It is mentioned that the selection process aims to reflect national strengths and priorities in bioinformatics and engage its national community. For readers who are not familiar with the bioinformatics community in the UK, it would be helpful to include a short, general description of the local bioinformatics landscape.

*Process overview*

 - Is "3. Setting up appropriate structures" referring to the Scientific Development Group or does it include other structures?

 - Which are the Working Groups that are mentioned in Figure 1 (you mention the Agriculture-related data WG)?

*Strategic Prioritization*

 - When you refer to "ELIXIR-UK", or "discussions within the Node", is this the same as "ELIXIR-UK Executive" in Figure 1 and the Executive Committee (http://www.elixir-uk.org/about-the-node)?

*Assessment criteria*

 - There is a mix here between the criteria for the ELIXIR Services (brought forward by the ELIXIR Nodes through the Service Delivery Plans) and the indicators which have been developed for the ELIXIR Core Data Resources. The latter focus on databases only and therefore won't be very helpful for training (for example). It would be good to make this explicit to avoid any potential confusion.

*Results of the assessment*

 - The list of assessment criteria is long and broad in scope. Is there any way of summarizing on which criteria the UK services are doing particularly well and on which criteria there can be improvement (or that were reasons for not including the services)?

 - Table 2: the services that are listed, seem to be UK-only. Certain are however broader collaborations (e.g. Ensembl, TeSS). Could this be made clear?

*Future activities and conclusions*

 - From the text, it seems that ELIXIR-UK is focusing on the identification of the ELIXIR UK Services. Is the UK node offering specific support (or other) to its services?

I have read this submission. I believe that I have an appropriate level of expertise to confirm that it is of an acceptable scientific standard.
